# Fecal microbiota transplantation confirmed that 919 Syrup reduced the ratio of erucamide to 5-AVAB in hippocampus to alleviate postpartum depression by regulating gut microbes

**DOI:** 10.3389/fimmu.2023.1203015

**Published:** 2023-05-24

**Authors:** Qiaoqi Zheng, Shusheng Wang, Xinyun Tian, Wen Liu, Pengfei Gao

**Affiliations:** ^1^ Department of Traditional Chinese Medicine, Jinshan Hospital, Fudan University, Shanghai, China; ^2^ Department of Radiology, Jinshan Hospital, Fudan University, Shanghai, China

**Keywords:** postpartum depression, 919 Syrup, fecal microbiota transplantation, erucamide, 5-aminovaleric acid betaine

## Abstract

**Background:**

Postpartum depression has a crucial impact on the physical and psychological comfort and the work of postnatal women, the growth and development of infants and mental health in adulthood. Finding a safe and effective anti-postnatal depression drug is currently an important research goal in this field.

**Methods:**

In this study, the forced swimming test (FST) and tail suspension test (TST) were used to evaluated the depressive behaviors of mice, and the changes of metabolites and intestinal microflora in mice with postpartum depression were examined through non-target metabolomics and 16S RNA sequencing respectively.

**Results:**

We found that traditional Chinese medicine compound 919 Syrup could alleviate postpartum depression in mice and inhibit the elevated erucamide level in depressive hippocampus. However, mice treated with antibiotics were not sensitive to the anti-postnatal depression effect of 919 Syrup, and the level of 5-aminovaleric acid betaine (5-AVAB) in their hippocampus was significantly decreased. Transplanting fecal microflora treated with 919 Syrup could effectively improve the depressive behaviors of mice, upregulate the level of gut-derived 5-AVAB in the hippocampus, and downregulate the level of erucamide. Erucamide was significantly negatively correlated with increased Bacteroides in intestine after 919 Syrup treatment or fecal transplantation, and significantly positively correlated with Ruminococcaceae UCG-014 which was increased in feces of mice with postpartum depression. The increase of Bacteroides, Lactobacillus, and Ruminiclostridium in intestine after fecal transplantation had a clearly positive correlation with 5-AVAB.

**Conclusion:**

In brief, 919 Syrup may downregulate the ratio of hippocampal metabolites erucamide to 5-AVAB by regulating intestinal flora to alleviate postpartum depression, laying a scientific foundation for future pathological research and development of therapeutic drugs for postpartum depression.

## 1 Introduction

Postpartum depression (PPD), one of the most common postnatal complications, is that a typical depressive episode occurs within one month after childbirth, usually manifested as persistent depression, irritability, anxiety, negativity, and loss of appetite. Most postpartum depression patients have a lack of willpower, and serious cases can lead to suicide and infanticide (1). In a 27-studies available for meta-analysis, the prevalence of PPD worldwide was estimated to arrange from 5.0% to 26.32% (2). In particular, the prevalence rate in China is as high as 21.4%, which is the highest in the world, significantly higher than 8.6% in the United States and 14.0% in Japan (2). Postpartum depression negatively impacts the mothers, with suicide accounting for approximately 20% of postpartum deaths (3), and has harmful effects on behavioural, emotional, and cognitive development of infant (4–8). Thus, understanding the underlying neurobiological mechanisms contributing to PPD and seeking corresponding therapeutic drugs for PPD play an imperative role on the health of mothers and offspring physically and mentally.

The gut-brain axis, also known as the bidirectional communication between the intestinal microbiota and the host’s central nervous system, has been the subject of a growing number of studies (9, 10). The ability of the gut microbes to secrete and upregulate essential proteins and metabolites involved in the release of neuropeptides and gut hormones, as well as govern the production of neurotransmitters and their precursors, making it an essential regulator of the gut-brain axis. (Such as serotonin, GABA, and tryptophan) (11–13). Similarly, our preliminary experiment found that it is possible that 919 Syrup could reshape the intestinal flora of post-natal depression mice to regulate the level of GABA in the hippocampus, thus treating post-natal depression (13). Here, the relationship between the therapeutic effect of 919 Syrup and intestinal flora and its potential mechanism will be further explored.

919 Syrup is a national patent compound Chinese medicine (Patent No. ZL 2020 1 1439679.6), the composition of which shown in **Table 1**. Previous clinical studies revealed that 919 Syrup could lessen PPD’s clinical signs. Our earlier animal studies demonstrated that 919 Syrup greatly reduced the anorexia symptoms and weight loss in postpartum stressed mice (14). Moreover, we further verified that 919 Syrup could treat postpartum depression through forced swimming test (FST) and tail suspension test (TST) (13). The level of erucamide in the hippocampus of postpartum depression mice increased (13). We also found that 919 Syrup could regulate intestinal flora while treating postpartum depression (13). People have gradually realized that there are inextricable links between traditional Chinese medicine (TCM) and intestinal microflora. After oral administration, Chinese medicine can interact with intestinal microbiota: 1. Chinese medicine can regulate the composition of intestinal microbiota; 2. TCM can regulate the metabolism of intestinal microflora; 3. Intestinal microbiota can transform traditional Chinese medicine compounds (15).

**Table 1:** The composition of 919 Syrup.

Name	Plant part	Botanical family	Proportion
Actinidia chinensis	Juice	Actinidiaceae	20g
Salvia miltiorrhiza	Roots and rhizomes	Lamiaceae	10g
Atractylodes macrocephala	Rhizome	Asteraceae	5g
Epimedium brevicornu	Plant	Berberidaceae	4g
Poria cocos	Sclerotium	Polyporaceae	4g
Schisandra chinensis	fruitage	Schisandraceae	5g
Elettaria cardamomum	Fruitage	Zingiberaceae	1.5g
Tangerine peel	Pericarp	Rutaceae	2.5g
Pseudostellaria heterophylla	Root tuber	Caryophyllaceae	5g
Bupleurum chinense	Plant	Apiaceae	2g

So, we proposed a feasible hypothesis that 919 Syrup may alleviate postpartum depression by reshaping intestinal flora and influencing hippocampal metabolism. In this work, we made every effort to show the connection between 919 Syrup and gut microorganisms during the treatment of PPD and to explain the molecular mechanism. We hope to open up a new window for the future development of therapeutic medicines for postpartum depression

## 2 Materials and methods

### 2.1 Mice

7-week-old male and female BALB/c mice were purchased from Shanghai Jihui Experimental Animal Feeding Co., Ltd (Shanghai, China) [SCXK (沪) 2017-0012] and raised as SPF in the Animal Laboratory Building of Fudan University’s Shanghai Public Health Clinical Center (Shanghai, China). Each set of five mice was kept in plastic cages (300 × 200 × 120 mm) with paper chips for bedding. Rooms were kept at a constant temperature (23 ± 3°C) and humidity (50% ± 10%), with simulated day/night cycles (12 hours each day, lights on at 8:00 am). Except during the immobilization phase, all mice had unrestricted access to regular food and autoclaved water throughout the experiment. The animal study was reviewed and approved by Shanghai Public Health Clinical Center Laboratory Animal Welfare (2021-A046-01). All authors are informed and agree.

### 2.2 Drug preparation

919 Syrup was produced by Shanghai Wanshicheng Pharmaceutical Co. Ltd. (Jinshan, Shanghai, China). According to the drug proportion in **Table 1**, five medicines, including Schisandra chinensis, Bupleurum chinense, Elettaria cardamomum, Tangerine peel and Atractylodes macrocephala, were put into a distillation pot, and three times of the weight of water was added for distillation. Then, the water was added to boil the distilled dregs and filter the juice. Salvia miltiorrhiza, Pseudostellaria heterophylla, Epimedium and Poria cocos were put into the frying pan, boiled twice with water, combined with the decoction twice, and filtered for juice. Then we mixed the above filtrate, adopted the low temperature and reduced pressure concentration method, concentrated to the liquid with the specific gravity of 1:1. After adding white granulated sugar at 30/10, the filtrate was boiled, dissolved and filtered. Then the filtrate with kiwi juice at 20/10 was mixed with distilled water. It was a liquid preparation with a concentration of 1.25g/ml. The traditional Chinese medicinal ingredients in 919 Syrup were published in the previous study (16).

Because our previous study concerning postpartum depression showed that the therapeutic effect was the best to attenuate depression-like behaviours in mice in the dose of 20 ml/kg (13), the intragastric dose of mice was 20 ml/kg in this study.

### 2.3 Postpartum restraint stress

**Figure 1:**
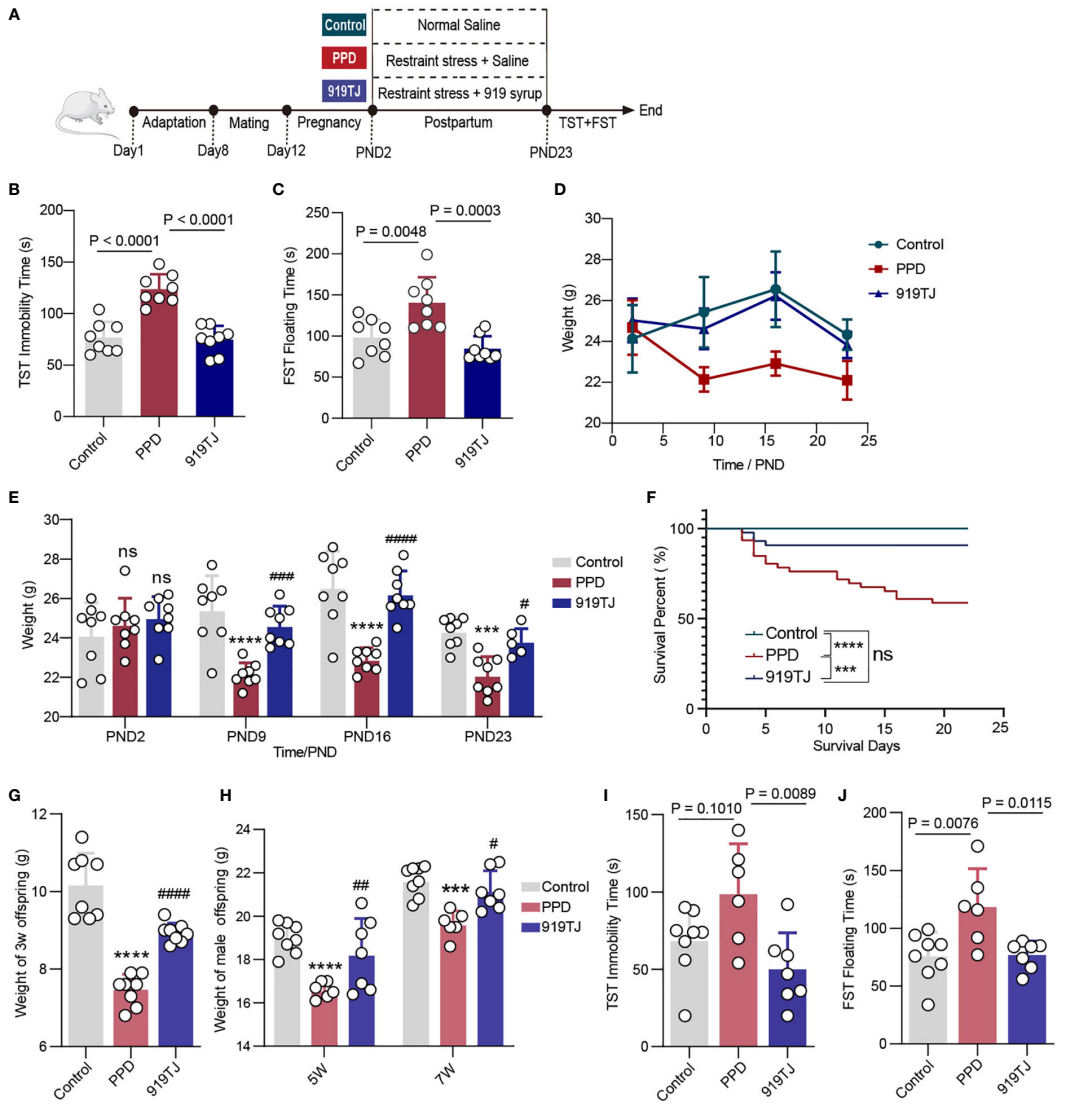
**(A)** Postpartum restrain stress process and experimental grouping; **(B)** Immobility time of behavioural experiment TST on PND24; **(C)** Floating time of behavioural experiment FST on PND25; **(D)** Line chart and **(E)** Histogram of postpartum weight changes in mice; **(F)** Survival curve of the new-born mice during three weeks after delivery; **(G)** Body weight of all of 3-week-old offspring, Control, n=8; PPD, n=8; 919TJ, n=8; **(H)** Body weight of male offspring at 5 and 7 weeks of age; **(I)** Immobility time of behavioural experiment TST of 7-week male offspring; **(J)** Floating time of behavioural experiment FST of 7-week male offspring. (Sample capacity: **(A, C-E)**: Control, n=8; PPD, n=8; 919TJ, n=8; **(H-J)**: Control, n=8; PPD, n=6; 919TJ, n=7. Statistics: **(E, G-H)**: *: PPD vs Control; #: 919TJ vs PPD; ***: P<0.001; ****: P<0.0001; #: P<0.05; ##: P<0.01; ###: P<0.001; ####: P<0.0001; n: P≥0.05).

After 7 days of adaptive feeding, the female and male mice were matched one by one and kept in each cage for 4 days until the negative plug was positive, and then they were kept separately in plastic cages. Delivery female mice (n = 30) were randomly divided into three groups with 10 mice in each group: Control group, PPD group and 919TJ group. We specified the birth date as the postpartum zero day (PND0). From PND2 to PND23, each female in PPD group and 919TJ group was separated from the cubs and subjected to restraint stress treatment for 3 hours from 13:00 to 16:00 every day. During this time, each mouse was fixed with a plastic holder. Before daily restraint stress treatment, the female mice in 919TJ group were given 20ml/kg 919 Syrup by gavage, and the other two groups were given the same dose of normal saline. The body weight and the number of offspring were recorded. (**Figure 1A**)

### 2.4 Antibiotic mixture treatment

**Figure 2:**
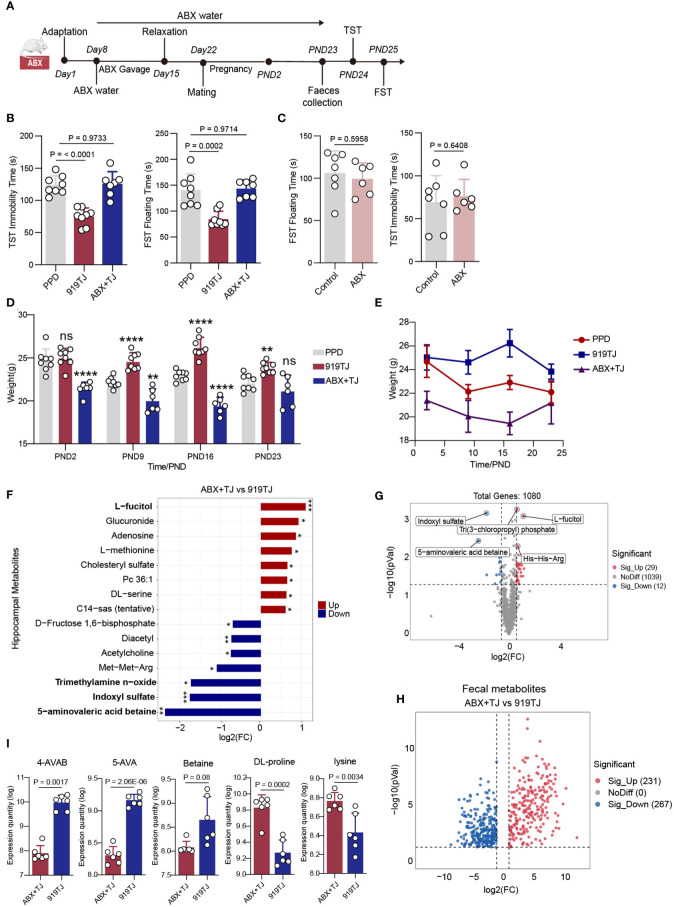
**(A)** Antibiotic treatment process; **(B)** Results of behavioural experiment FST and TST of PPD, 919TJ and ABX+TJ group; PPD, n=8; 919TJ, n=8; ABX+TJ, n=7; **(C)** Results of behavioural experiment of Control vs ABX; Control, n=7; ABX, n=6; **(D)** Histogram and **(E)** line chart of postpartum weight changes in mice; PPD, n=8; 919TJ, n=8; ABX+TJ, n=6; *: comparison of other groups with PPD, **: P<0.01; ****: P<0.0001, n: P≥0.05; **(F)** Differential hippocampal metabolites of ABX+TJ vs 919TJ, discriminative variants were identified on the basis of a VIP value≥1, P value<0.05, log2(FC) >|1|; **(G)** Volcanic map of hippocampal metabolites in ABX+TJ vs 919TJ, 29 was significantly upregulated and 12 was downregulated, Fold change≥|1.5|, P value<0.05; **(H)** Volcanic map of deferential faecal metabolites of ABX+TJ vs 919TJ, Fold change≥|2|, P value<0.05; **(I)** Expression quantity of 4-AVAB, 5-AVA, betaine, DL-proline and lysine in faeces of mice.

According to the procedures outlined by Vuong, H., et al. (17), seven-week-old SPF mice were given an oral mixture of neomycin (100 mg/kg), metronidazole (100 mg/kg), and vancomycin (50 mg/kg) twice daily (08:00 and 17:00) for one week. Ad libitum ampicillin (1 mg/ml) was given in water at the same moment. Other non-antibiotic treated mice were given the same dose of normal saline by gavage. After one-week antibiotic mixture gavage, mice were maintained on antibiotic drinking water (1 mg/ml ampicillin, 1 mg/ml neomycin, and 0.5 mg/ml vancomycin) until behavioural tests. Other non-antibiotic treated mice were given normal water. Mice were given a one-week rest period before to mating to reduce the daily stress of oral gavage of antibiotic combination. After that, mice were matched and mated, where conception could take up to ten days, and then were separated after seeing the copulation plugs (**Figure 2A**).

### 2.5 Fecal microbiota transplantation

**Figure 3:**
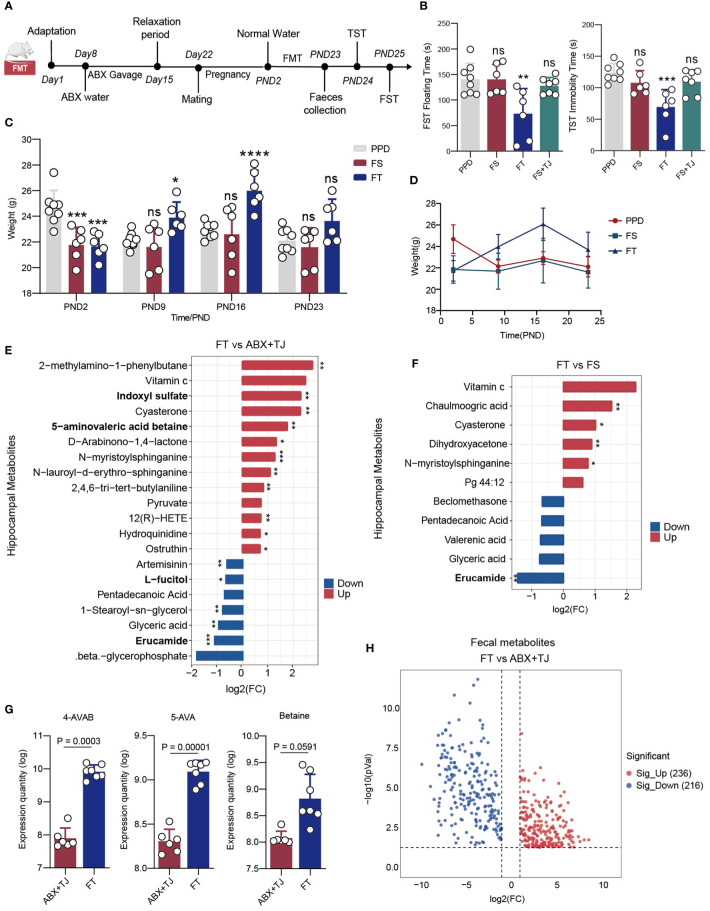
**(A)** Faecal microbe transplantation process; **(B)** Results of behavioural experiment FST and TST of mice; **(C)** Histogram and **(D)** line chart of postpartum weight changes in mice; PPD, n=8; FS, n=6; FT, n=6; FS+TJ, n=7; **(E)** Differential hippocampal metabolites of FT vs ABX+TJ and **(F)** of FT vs FS, discriminative variants were identified on the basis of a VIP value≥1, Fold change≥|1.5|, P value<0.05; **(G)** Expression quantity of 4-AVAB, 5-AVA and betaine in faeces of mice; **(H)** Volcanic map of deferential faecal metabolites of FT vs ABX+TJ, VIP value≥1, Fold change≥|2|, P value<0.05. B, C: *: comparison of other groups with PPD, *: P<0.05; **: P<0.01; ***: P<0.001; ****: P<0.0001, n: P≥0.05).

The method of faecal microbiota transplantation (FMT) refers to the research of Cignarella, F., etc (18). Up until delivery, BALB/c female mice received a combination of antibiotics to deplete their gut flora before being employed as FMT recipients. After production, recipients’ antibiotic-infused water was switched out for regular water. Faeces from donor mice were given to recipient mice by oral gavage daily for three weeks as part of the transplant procedure. In a nutshell, each donor’s faeces pellets were collected every day, and each 6 gram was dissolved in 1 mL of sterile PBS, filtered through a 100-m sterile filter screen, and then the soluble fraction was given to the recipients via oral gavage (200μl/each). It should be noted that the date of collection of faeces by the providers corresponds to the date of post-natal of the recipients. For example, the faeces of PPD mice in PND5 were taken and orally administrated to FS mice in PND5 to ensure that the daily changes in intestinal flora of FS and FS+TJ mice were consistent with those in PPD mice, as were the FT and 919TJ groups. (**Figure 3A**)

### 2.6 Experimental grouping

BALB/c female mice (n=80) were randomly divided into 8 groups: Control, postpartum depression (PPD), 919 Syrup treatment (919TJ), antibiotic mixture treatment (ABX), antibiotic mixture and 919 Syrup treatment (ABX+TJ), feces transplantation from PPD (FS), feces transplantation from 919TJ (FT) and feces transplantation from PPD+919 Syrup treatment (FS+TJ).

The mice in ABX, ABX+TJ, FS, FT and FS+TJ received the antibiotic mixture treatment descripted above, among which, the antibiotic drinking water of FS, FT and FS+TJ was replaced with normal drinking water after delivery, making them as the recipients of FMT. Except for the mice in Control, the others were subjected to postpartum restraint stress for three weeks after giving birth. One hour before the daily postpartum restraint stress, mice in 919TJ, ABX+TJ and FS+TJ were given 919 Syrup by gavage (20ml/kg), and the other groups were given the same dose of normal saline. In addition, the fecal suspension collected from PPD mice was orally administered to FS and FS+TJ mice and that from 919TJ mice was given to FT mice by gavage once a day (200µl/each).

### 2.7 Tail suspension test

The tail suspension test (TST) was performed at 17: 00-19: 00 on PND24 according to the description by Dunn and Swiergiel (19). The mouse tail was taped in place 30 cm above the table in a visual isolation zone for six minutes. The mouse tail tip and the tape were separated by about 2 cm. The first two minutes of the six-minute trial session were considered the adaption phase, and the remaining four minutes were used to measure the immobility time. When the mouse’s hind paw was immobile and hung passively, it was considered immobile.

### 2.8 Forced swimming test

The forced swimming test (FST) was used at 17: 00-19: 00 on PND25 to estimate the depression-related behavior. The mice were put inside a plexiglass cylinder that was 200 mm high by 140 mm in diameter and filled with water, which was 21°C or slightly warmer, to a height of 10 cm, preventing the mice from climbing out or touching the bottom for 6 minutes, of which recorded the immobility time of mice for the last 4 minutes. A mouse was considered to be immobile if it floated in the water and made no motion.

### 2.9 16S rRNA gene sequencing

According to the method we described earlier (13), around 12 a.m. on PND23, faecal samples were obtained from mice and immediately frozen in liquid nitrogen. In simple terms, the CTAB/SDS technique was used to extract whole genome DNA, and the purity and concentration of the DNA were determined. Polymerase chain reaction (PCR) was used to amplify a specified V3-V4 variable area for sequencing utilizing barcode-specific primers and high-fidelity DNA polymerase. The PCR results were identified using 2% agarose gel electrophoresis, and the target fragment was gel-cut and recovered. The sequencing libraries was created and the library quality was evaluated. Lastly, the library was sequenced on an Illumina HiSeq2500, yielding 300bp paired-end reads.

### 2.10 Untargeted metabolomics

Around 12 a.m. on PND23, faeces were collected and rapidly frozen in liquid nitrogen. On PND23, mice were terminated by intraperitoneal injection of 2.5% tribromoethanol in normal saline. The hippocampus was surgically removed from each mouse and instantly frozen in liquid nitrogen following dissection. By combining 10 mL of each sample, quality control (QC) samples were made, and they were examined alongside the other samples to evaluate the consistency and repeatability of instrument analysis. At Shanghai Applied Protein Technology Co., Ltd., a UHPLC (1290 Infinity LC, Agilent Technologies) linked to a quadrupole time-of-flight (AB Sciex TripleTOF 6600) was used for analysis. Samples were analysed using a 2.1 mm × 100 mm ACQUIY UPLC BEH 1.7 µm column (Waters, Ireland) for HILIC separation. The mobile phase in both ESI positive and negative modes contained A=25 mM ammonium acetate and 25 mM ammonium hydroxide in water, and B=acetonitrile. The gradient was 95% B for 0.5 minute, then linearly reduced to 65% in 6.5 minutes, 40% in 1 minute and held for 1 minute, and 95% in 0.1 minute, with a 3 minutes re-equilibration period.

### 2.11 Correlation analysis

To begin with, the relative abundances (LefSe LDA>2 and p value<0.05) of bacterial populations with significant differences at the genus level obtained through 16S rDNA amplification sequencing analysis in all experimental samples, as well as the expression levels of significantly different metabolites (VIP>1) and p-value<0.05 for t-test obtained through metabolomics analysis, were collated in a table as input files for subsequent analysis.

### 2.12 Statistical analysis

#### 2.12.1 16S rRNA gene sequencing

Statistical Analysis of Metagenomic Profiles (STAMP v2.1.3) (20) was used to confirm differences in the abundance of individual taxonomies between each group. LefSe was used to conduct quantitative analyses of biomarkers in various groups. This method was developed to analyse data with a large number of species compared with samples, and to provide biological class explanations to establish statistical significance, biological consistency, and effect-size estimation for the predicted biomarkers. Anosim and adonis were used to identify differences in the microbial communities between the two groups using the Bray-Curtis dissimilarity distance matrices. Anosim analysis is a non-parametric test based on the Bray-Curtis algorithm that determines whether differences between groups are significantly greater than differences within groups in order to determine whether the grouping is meaningful. Adonis is also known as nonparametric MANOVA or permutational MANOVA. The substitution test is used to determine the statistical significance of this grouping based on the explanatory degree of different grouping factors to sample differences.

#### 2.12.2 Untargeted metabolomics

The sum-normalized data were then submitted to multivariate data analysis using the R package (ropls), which included Pareto-scaled principal component analysis (PCA) and orthogonal partial least-squares discriminant analysis (OPLS-DA). The model’s stability was assessed using response permutation testing and 7-fold cross-validation. Each variable in the OPLS-DA model had its variable importance in the projection (VIP) value computed to show how it contributed to classification. The significance of differences between two sets of independent samples was assessed using Student’s t test. Significantly altered metabolites were screened using VIP > 1 and a p value < 0.05. The correlation between two variables was examined using Pearson’s correlation technique.

#### 2.12.3 Correlation analysis

Significantly different-expressed genes and metabolites were Z-score adjusted and combined into a single matrix. R Version 3.4.2 (R-Foundation, Vienna, Austria) was used to compute the correlation coefficient of each molecule in the matrix taking into account the non-normal distribution of the original data.

#### 2.12.4 Behavioural experiments, and etc.

Every trial was performed at least three times, and the standard deviations were computed. In order to analyse the data, GraphPad Prism 8.0 was used (La Jolla, CA, USA). Following a one-way analysis of variance (ANOVA), a Bonferroni post-test was used to assess the statistical significance of the results between groups. Offspring Survival data’s between-group statistical relevance was assessed using the survival curve. Statistical significance was defined as a p-value < 0.05.

## 3 Results

### 3.1 919 Syrup is effective in alleviating postpartum depression

After suffering postpartum restraint stress, the weight of PPD mice decreased obviously compared with Control, however, it raised significantly in 919TJ group. (**Figures 1D, E**) The immobility time of TST and the floating time of FST both significantly increased in PPD group compared with Control group, which were dramatically reduced following intragastric injection of 919 Syrup in 919TJ group. (**Figures 1B, C**) Mothers with postpartum depression are more prone to infanticide than the normal (21, 22), for which we kept track of how long the offspring survived and how many of them perished from the mother mice’s bite from the 2nd to 23nd day after birth. The survival rate of PPD group’s offspring greatly declined compared with Control one, whereas that of the 919TJ group significantly rose compared with PPD group. (**Figure 1F**) The female mice in the 919TJ also ate their new-borns in the first six days following delivery, but it had a very low probability of this behaviour and thereafter vanished. (**Figure 1F**)

### 3.2 The effect of 919 Syrup on progeny in the treatment of PPD

What’s more, we measured the weight of offspring at the age of three weeks, five weeks and seven weeks respectively, finding that the weight of those whose mother suffered from PPD was significantly reduced, while 919 Syrup treatment could promote the growth and development of offspring, effectively increasing their weight. (**Figures 1G, H**) At 7 weeks of age, which is comparable to adolescence in humans (23), we decided to undertake a behavioral evaluation of depression in offspring mice. However, as female mice are more susceptible to the abrupt hormonal changes associated with puberty, we focused our research on depressed behavior in male pups. The male progeny of mothers with PPD seemed to be more sensitive to adolescent depression, which was verified by FST and TST. (**Figures 1I, J**) After the mother’s reception of 919 Syrup treatment, the immobility time of their offspring in TST and the floating time in FST were both significantly declined compared with that of PPD group. (**Figures 1I, J**)

### 3.3 The consumption of 919 Syrup downregulates erucamide in hippocampus of mice

**Figure 4:**
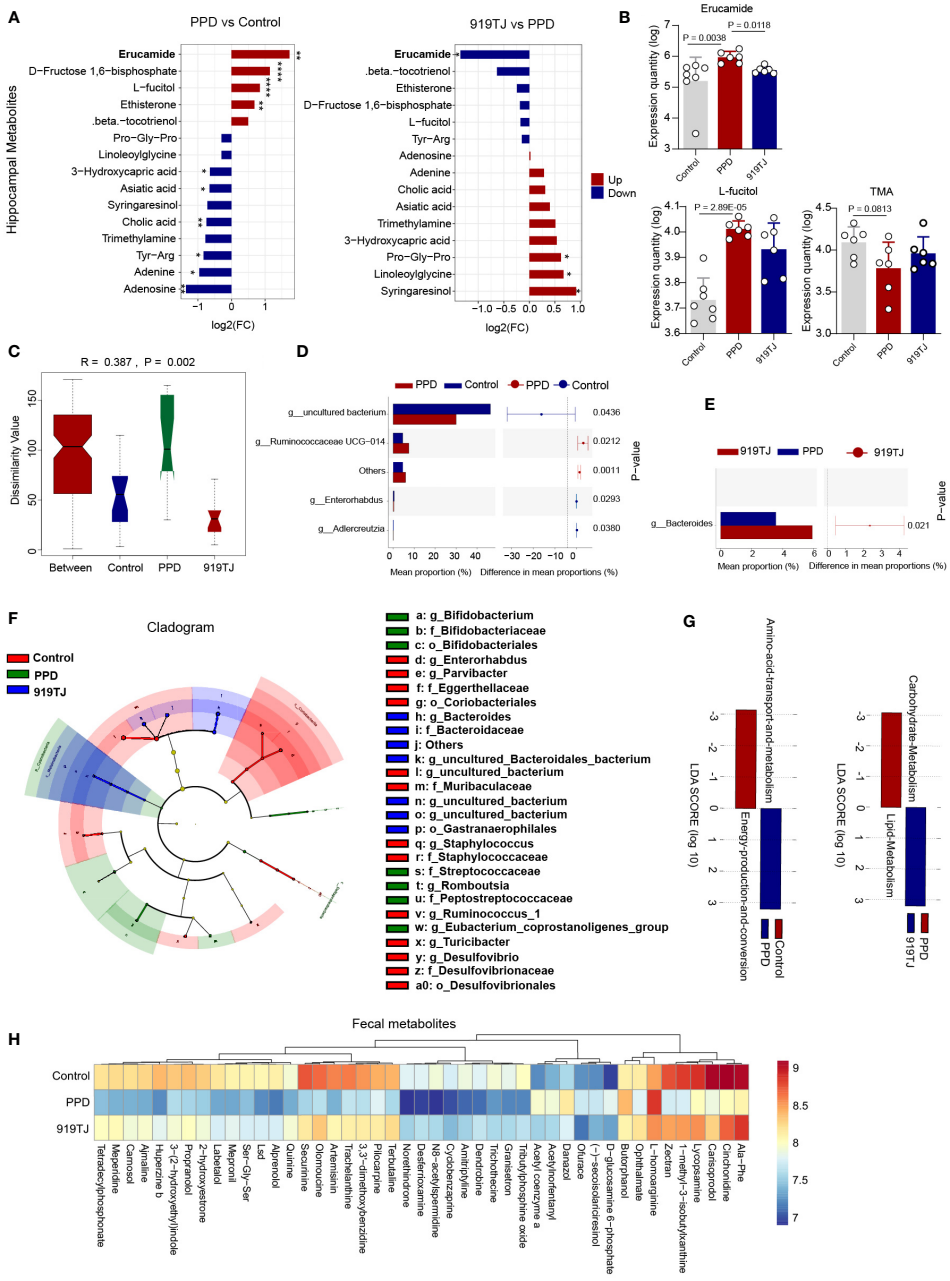
**(A)** Fifteen differential hippocampal metabolites in PPD vs Control and 919TJ vs PPD; discriminative variants were identified on the basis of a VIP value ≥1, P value <0.05, log2(FC) >|1|; *, P<0.05; **, P<0.01; ****, P<0.0001. **(B)** Expression quantity of Erucamide, L-fucitol and TMA in hippocampus of mice; **(C)** Anosim of beta diversity analysis of faecal flora; **(D)** STAMP analysis of faecal flora of PPD and Control mice (t test P value<0.05); **(E)** STAMP analysis of faecal flora of PPD and 919TJ mice (t test P value<0.05); **(F)** LefSe analysis of faecal flora of mice; **(G)** Analysis of KEGG function of different faecal microflora; **(H)** Faecal metabolites of mice, VIP value≥1, Fold change≥|2|, P value<0.05. (Sample capacity: Control, n=7; PPD, n=6; 919TJ, n=6).

Four metabolites were considerably elevated and nine metabolites were significantly downregulated in the hippocampus of PPD mice compared with controls. (**Figure 4A**) Compared with PPD group, 919TJ group significantly downregulated 2 metabolites and upregulated 4 metabolites. (**Figure 4A**) Among them, erucamide, which is also our focus, was evaluated in hippocampus of PPD mice (P=0.0038), consistent with our previous study (13), and decreased significantly in 919TJ mice (P=0.0118). (**Figure 4B**) L-fucitol was apparently increased after postpartum restraint stress (P=2.89E-05) but not reduced by 919 Syrup treatment. (**Figure 4B**) Trimethylamine was downregulated in PPD status (P=0.0813) but not reversed in 919TJ mice (**Figure 4B**).

### 3.4 Changes of gut bacteria in PPD and 919TJ mice

The mice of Control, PPD, and 919TJ groups had significantly different intestinal microbiota, according to beta diversity analyses. (**Figure 4C**) The results of beta diversity analysis are shown in **Table 2**. We then used STAMP analysis (**Figures 4D, E**) and LefSe analysis (**Figure 4F**) to compare the differences in the composition of intestinal microflora in mice. Compared with the Control group, the intestinal microbiota of mice in the PPD group was significantly increased in g_Ruminococcaceae UCG-014 (P=0.0212), while g_Errorhabdus (P=0.0293), g_Adlercreutzia (P=0.038) and g_uncultured_Bacterium (P=0.0436) was significantly decreased. (**Figure 4D**) Compared with PPD group, the abundance of g_Bacteroides (P=0.021) in 919TJ group increased significantly. (**Figure 4E**) The dominant strains in PPD group mainly include g_Bifidobacterium, o_Bifidobacteriaceae, f_Bacteroidaceae and etc. (**Figure 4F**) In the 919TJ group, the dominant bacteria mainly include o_Gastranaerophiles, f_Bacteroidaceae, g_Basteroides and etc. (**Figure 4F**) KEGG function analysis was performed on the different bacterial populations between groups. (**Figure 4G**) Compared with Control group, 198 kinds of stool metabolites in PPD group changed, of which 88 were upregulated and 110 were significantly downregulated. The 919TJ group contained 66 different types of stool metabolites compared with the PPD group, 44 of which showed substantially upregulated and 22 significantly downregulated expression. Some of the faecal differential metabolites are shown here (**Figure 4H**).

**Table 2:** The results of beta diversity analysis on faecal flora.

Groups	Beta diversity analysis	R value	P value
Control_vs_PPD_vs_TJ919	Adonis	0.237773139	0.001
	Anosim	0.387091503	0.002
TJ919_vs_ABX+TJ_vs_FT	Adonis	0.801064407	0.001
	Anosim	0.80620915	0.001
ABX+TJ_vs_FS_vs_FT	Adonis	0.75954452	0.001
	Anosim	0.633660131	0.001
ABX+TJ_vs_FT_vs_FS+TJ	Adonis	0.741298315	0.001
	Anosim	0.642923097	0.001
PPD_vs_Control	Adonis	0.151176802	0.003
	Anosim	0.255813953	0.023
TJ919_vs_PPD	Adonis	0.11604696	0.063
	Anosim	0.187037037	0.04
ABX+TJ_vs_TJ919	Adonis	0.5779752	0.001
	Anosim	1	0.001
FT_vs_FS	Adonis	0.060915407	0.469
	Anosim	-0.036241319	0.684
FT_vs_ABX+TJ	Adonis	0.594446025	0.001
	Anosim	1	0.001
FS_vs_ABX+TJ	Adonis	0.565726839	0.001
	Anosim	1	0.001
FT_vs_FS+TJ	Adonis	0.103632328	0.062
	Anosim	0.103035366	0.102

### 3.5 The therapeutic effect of 919 Syrup on postpartum depression depends on the existence of intestinal floras

We had constructed a relatively Intestinal-Bacteria-Free (IBF) animal model of postpartum depression by antibiotic treatment. (**Figure 2A**) The depressive behaviors of mice were unaffected by simply removing the gut flora, and there was no discernible difference between the IBF mice in FST and TST and the Control group in terms of their immobility time. (**Figure 2C**) Through this method, we first ruled out the effect of using antibiotic mixtures alone on postpartum behavior in mice. However, when IBF mice with postpartum depression received 919 Syrup treatment, the immobility time in FST and TST was not different from that in the PPD group and was significantly longer than that in the 919 Syrup treatment group. Intestinal flora is necessary for 919 Syrup to be effective in treating postpartum depression symptoms. (**Figure 2B**) What’s more, when the intestinal tract was sterile, the weight loss associated with postpartum depression cannot be reversed by the treatment of 919 Syrup (**Figures 2D, E**).

### 3.6 Antibiotic treatment reduces gut-derived 5-AVAB and its downstream metabolite TMAO in the hippocampus of postnatal depression mice

In the hippocampus of ABX+TJ mice, compared with 919TJ group, 29 metabolites were significantly up-regulated and 12 metabolites were significantly down-regulated. (**Figure 2G**) Among them, 5-aminovaleric acid betaine (5-AVAB), trimethylamine n-oxide (TMAO) and Indoxyl sulfate (IS) were significantly decreased in hippocampus of mice in ABX+TJ compared with 919TJ, while L-fucitol was significantly upregulated after antibiotic treatment. (**Figure 2F**) Compared with 919TJ group, 231 metabolites were significantly upregulated and 267 metabolites were significantly downregulated in the feces of ABX+TJ mice. (**Figure 2H**) Among them, DL-proline and lysine (Lys), the raw materials for synthesizing 5-AVAB, have increased significantly. (**Figure 2I**) 4-aminovaleric acid betaine (4-AVAB), 5-aminovaleric acid (5-AVA) and Betaine, the precursors for synthesizing 5-AVAB, were significantly reduced (**Figure 2I**).

### 3.7 Alterations of intestinal bacteria after Antibiotic treatment

**Figure 5:**
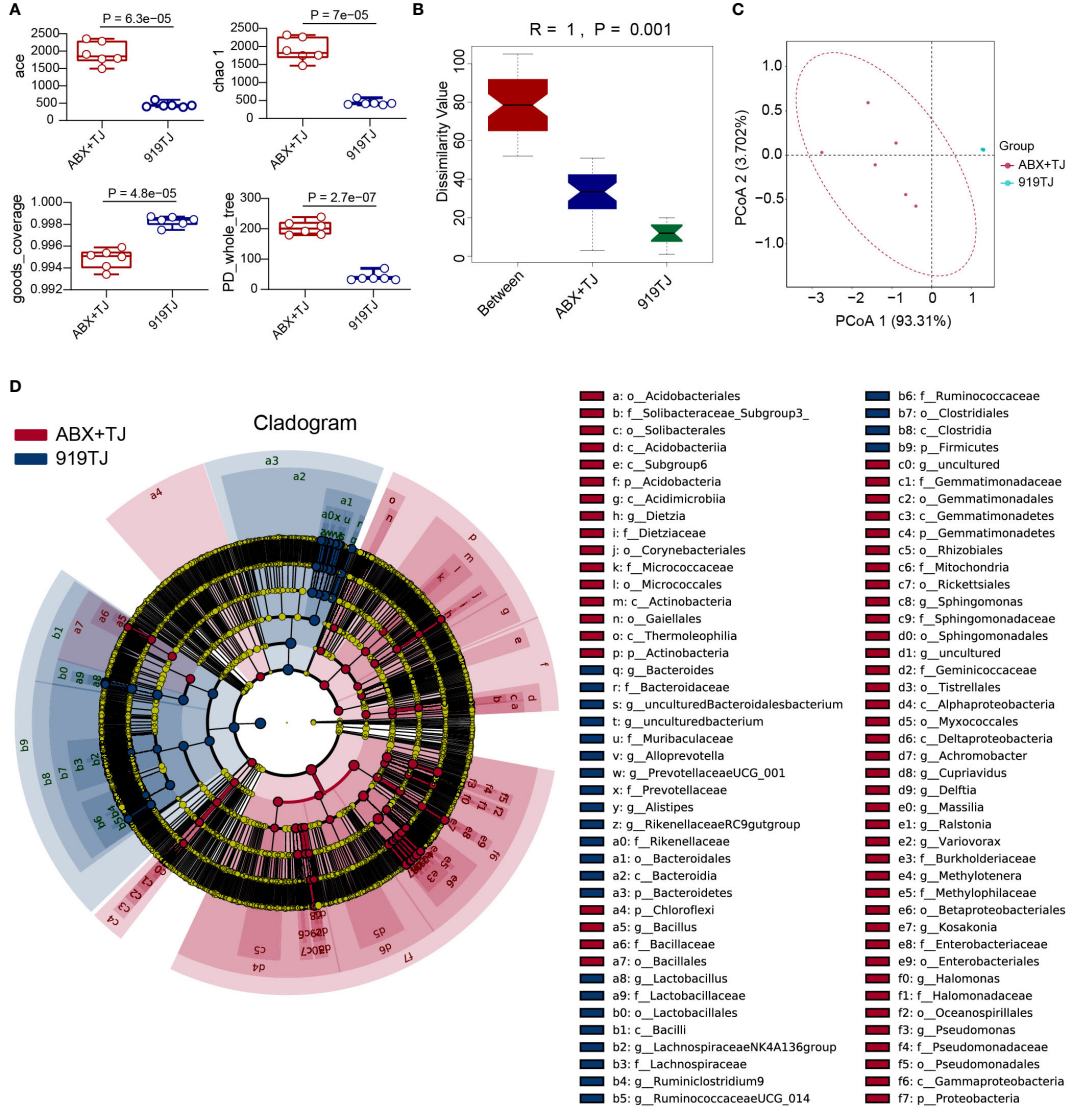
**(A)** Alpha diversity analysis of faecal flora of ABX+TJ vs 919TJ; **(B)** Anosim of beta diversity analysis of faecal flora; **(C)** PCoA analysis of faecal flora; **(D)** LefSe analysis of faecal flora of mice, LDA Scores≥3.5, P value<0.05. (Sample capacity: ABX+TJ, n=6; 919TJ, n=6).

When compared with 919TJ, several indications of Alpha diversity study revealed considerable variations in intestinal microflora during antibiotic treatment. (**Figure 5A**) Beta diversity analysis also showed a significant difference in intestinal microflora among mice of ABX+TJ and 919TJ group. (R=1, P=0.001) (**Figures 5B, C**) The results of beta diversity analysis are shown in **Table 2**. After antibiotic treatment, 68 intestinal floras were significantly upregulated and 39 were downregulated. (**Figure 5D**) The upregulated intestinal microflora in group ABX+TJ include p_Proteobacteria, o_Pseudomonadales, f_Pseudomonadaceae, g_Pseudomonas, g_Achromobacter and etc. (**Figure 5D**) o_Bacteroidales,c_Bacteroidia,p_ Bacteroidetes,f_ Muribaculaceae,p_ Firmicutes,g_ Lactobacillus,f_ Lactobacillaceae and et al. were downregulated (**Figure 5D**).

### 3.8 Anti-postpartum depression effect of fecal microbiota transplantation

So far, we have confirmed that the anti-postpartum depression effect of 919 Syrup was closely related to intestinal bacteria in mice. However, there are many kinds of connections. Here we put forward two situations with the greatest possibility: 1. Intestinal flora composition is regulated by 919 Syrup, which also affects metabolites and their actions; 2. Certain components of the traditional Chinese medicine compound can reach the blood and actually function as a therapeutic agent after the digestion and metabolism of intestinal flora, whereas the components cannot enter the blood when the digestive tract is sterile. Therefore, we designed three groups of fecal microbiota transplantation to verify these two hypotheses. (**Figure 3A**) First of all, during the three weeks after delivery, FS mice were given fecal suspension from PPD mice every day. The results showed that the immobility time of FS mice in FST and TST tests was not different from that of PPD mice. (**Figure 3B**) Then, on the basis of FS group, FS+TJ group mice were given the same dose of 919 Syrup as 919 TJ group every day. The behavioral results were consistent with FS group. (**Figure 3B**) However, when FT mice were given fecal suspension from 919TJ mice by gavage, the behavioral test results showed that the duration of immobility in FST and TST was significantly shorter than that in PPD group. (**Figure 3B**) Consistent with this, FT group mice showed better weight recovery effect in PND9 and PND16 compared with FS group (**Figures 3C, D**).

### 3.9 Transplanting feces from 919TJ group upregulates the levels of gut-derived 5-AVAB and downregulates erucamide in hippocampus of mice

Having received fecal transplantation from 919TJ group, 5-AVAB in the hippocampus of FT mice was significantly upregulated, while erucamide and L-fucitol were significantly reduced. (**Figure 3E**) Transplanting feces of 919TJ group instead of PPD could downregulated the level of erucamide in hippocampus of mice. (**Figure 3F**) In the gut of FT mice, 236 kinds of stool metabolites were upregulated and 216 were significantly downregulated, compared with ABX+TJ mice. (**Figure 3H**) Among them, 4-AVAB, 5-AVA and Betaine, the precursors for synthesizing 5-AVAB, were significantly upregulated (**Figure 3G**).

**Figure 6:**
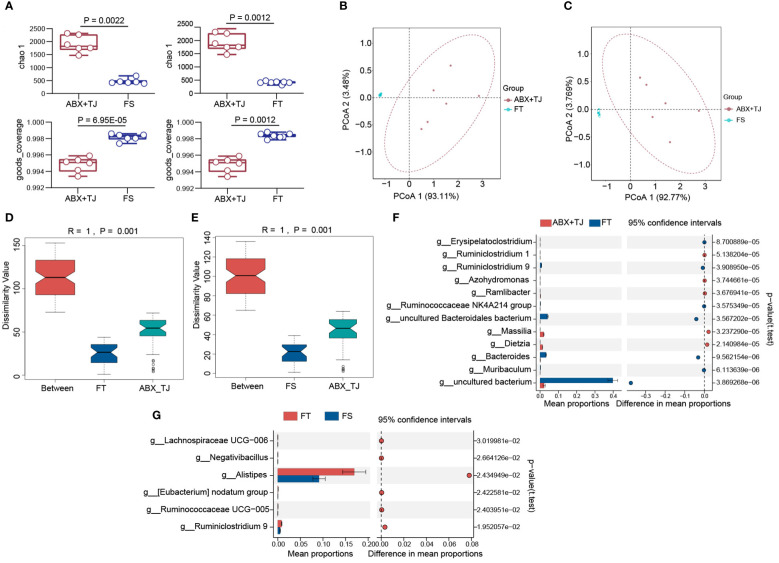
**(A)** Alpha diversity analysis of faecal flora of FS vs ABX+TJ and FT vs ABX+TJ; **(B)** PCoA analysis of faecal flora of FT vs ABX+TJ; **(C)** PCoA analysis of faecal flora of FS vs ABX+TJ; **(D)** Anosim of beta diversity analysis of faecal flora of FT vs ABX+TJ, R=1, P=0.001; **(E)** Anosim of beta diversity analysis of faecal flora of FS vs ABX+TJ, R=1, P=0.001; **(F)** STAMP analysis of faecal flora of FT and ABX+TJ mice (t test); **(G)** STAMP analysis of faecal flora of FS and ABX+TJ mice (t test). (Sample capacity: ABX+TJ, n=6; FS, n=6; FT, n=7).

Indexes of Alpha diversity analysis showed significant differences in intestinal microflora after fecal transplantation. (**Figure 6A**) Beta diversity analysis also showed a significant effect on intestinal microflora recovery after fecal transplantation. (R=1, P=0.001) (**Figures 6B-E**) STAMP analysis found that the transplantation of feces from mice of 919TJ group mainly restored the following levels of intestinal flora: g_Bacteroides, g_uncultured Bacteroidales bacterium, g_uncultured bacterium and g_ Ruminiclostridium 9, same as 919TJ group. (**Figure 6F**) Compared to FS group, g_ Ruminiclostridium 9 was also increased significantly (**Figure 6G**).

### 3.10 Correlation analysis of hippocampal metabolites and intestinal floras

**Figure 7:**
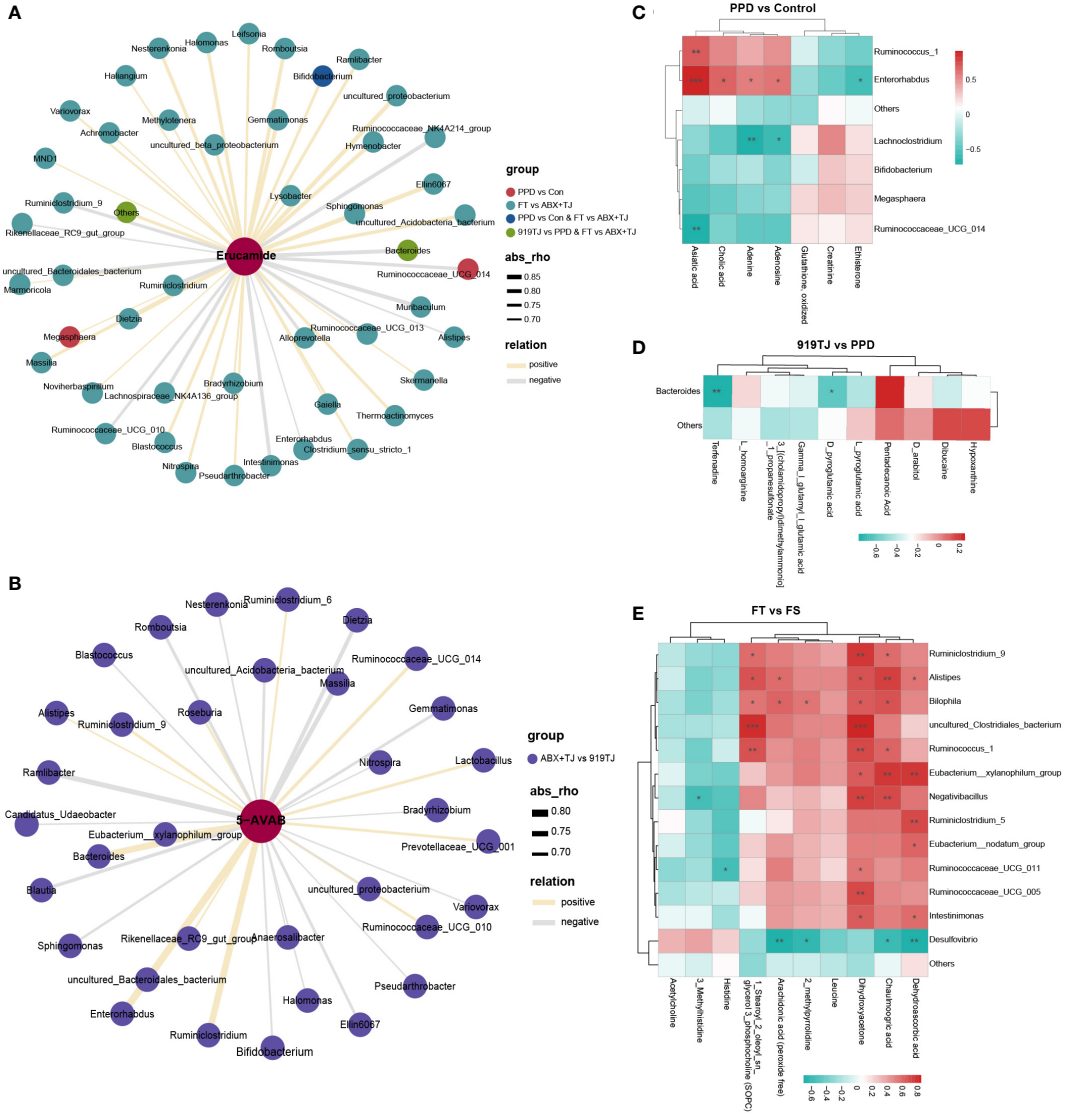
**(A)** Network diagram of correlation analysis between erucamide and differential faecal microflora and **(B)** Network diagram of correlation analysis between 5-AVAB and differential faecal microflora of ABX+TJ vs 919TJ; colour of dot represents different group, the width of the line represents the correlation coefficient, yellow line represents a positive correlation, and grey line represents a negative correlation, coefficient≥|0.6|, P value<0.05; **(C)** Correlation analysis between differential metabolites in hippocampus and differential faecal microflora of PPD vs Control, of **(D)** 919TJ vs PPD and of **(E)** FT vs FS; the red grid represents a positive correlation, while the green grid represents a negative correlation. (C-E: *: P<0.05; **: P<0.01; ***: P<0.001).

Next, we tried to find a potential relationship between hippocampal metabolites and intestinal microbes through correlation analysis. The upregulation of erucamide in the hippocampus of PPD mice may be related to Megasphaera, Bifidobacterium, and Ruminococcaceae_UCG_014. (**Figure 7A**) 919 Syrup may downregulate the level of erucamide in the hippocampus by regulating Bacteroides. (**Figure 7A**) The downregulation of erucamide levels after 919TJ fecal transplantation may be also related to Bacteroides. (**Figure 7A**)

After antibiotic treatment, the level of 5-AVAB in the hippocampus of mice was significantly decreased, which may be related to the decrease of Lactobacillus, Ruminiclostridium, and Bacteroides. (**Figure 7B**) Transplantation of 919TJ group feces could restore these bacteria and increase the level of 5-AVAB in the hippocampus. The correlation analysis results between other hippocampal metabolites and intestinal microflora are shown in **Figures 7C-E** and Supplementary materials.

## 4 Discussion

Postpartum depression is widely documented to have a significant influence on the physical and mental health of postpartum moms and their infants. It is important to find a safe and effective anti-postnatal depression drug. In this study, 919 Syrup was demonstrated to alleviate postpartum depression, promote the growth and development of offspring, and improve the depressive behaviors of male progeny.

It has been confirmed that the mechanism of 919 Syrup in treating postpartum depression is related with intestinal flora of mice. Researches have shown that gut microbiota has a direct impact on emotional behaviors, and there is a difference in anxiety behaviors between Germ Free (GF) rats, mice and other animals with normal gut microbiota (24–27). The colonization of normal intestinal flora in GF animals has been shown to improve behavioral differences (25, 26, 28). Many human and animal studies have reported that probiotics could reduce anxiety and depression (29–33). Although there are many convincing evidences that intestinal microbiota is related to emotional behaviors, we do not fully understand its mechanism and clinical relevance. In the process of interaction between TCM and intestinal floras, two types of metabolites can be produced: metabolites of intestinal microbiota (food and host sources) and TCM compounds transformed by intestinal microbiota (15). Therefore, we proposed two possible mechanisms for 919 Syrup to rely on intestinal microorganisms in the treatment of postpartum depression. FMT has demonstrated that the way 919 Syrup functions is related to the intestinal flora itself, but not to the TCM compounds transformed by intestinal microbiota. On this basis, we further search for key intestinal microflora and their metabolites for the treatment of PPD.

We discovered that the erucamide in the hippocampus of PPD mice significantly rose, which was consistent with our earlier study findings (13). The experimental results were repeatable. After 919 Syrup treatment, erucamide in the hippocampus of mice decreased significantly. Erucamide is a fatty acid amide that regulates physiological activities through receptors, such as angiogenesis (34) and water balance (35). The plasma level of erucamide in pig body organs was 3 ng/g, while the lung, kidney, liver, and brain levels were 12, 2.5, 1.0, and 0.5 ng/g, respectively (35). The concentration in brain was the lowest, and it was known to exist in cerebrospinal fluid. The slight fluctuation of brain level might have an unpredictable amplification effect (35). Li et al. compared erucamide to the traditional antidepressant fluoxetine and found that it had similar antianxiety and antidepressant effects in the mouse model (36). However, in our study, the level of erucamide in the hippocampus of mice with postpartum depression after treatment with 919 Syrup, a traditional Chinese medicine compound, decreased significantly. In mice with PPD, erucamide seems to play a different role. In our study, the level of erucamide in the hippocampus of FT group is significantly lower than that of FS and ABX+TJ group, suggesting that erucamide in the hippocampus originates from the intestine, and 919 Syrup may mediate the reduction of erucamide to alleviate PPD by regulating intestinal microflora.

However, there was no statistically significant change in the level of erucamide between ABX+TJ and 919TJ mice in the hippocampus. Compared with 919TJ group, the level of 5-AVAB and its metabolite TMAO in the hippocampus of ABX+TJ mice were significantly reduced. 5-AVAB is a trimethyl compound, and its sources mainly include three pathways: endogenous synthesis, dietary sources, and gut microbiota (37). There is strong evidence that this metabolite is at least largely generated by intestinal microflora (17, 38–41), as indicated by certain research findings that there is a positive association between intestinal microbiota and 5-AVAB abundance (17, 39, 40), which is consistent with our research results. Several significant bacteria, such as Bifidobacteria and Coriobacteriaceae, whose abundance is linked to the synthesis of 5-AVAB, are present in the typical gut microbiome (39). One of the most likely precursors in the biosynthesis of 5-AVAB is 5-AVA. There is enough proof to conclude that L-lysine can be used as the starting material for Pseudomonas bacteria to generate 5-AVA (42, 43). In addition, Enterococcus faecalis could utilize the DavB and DavA enzyme pathways to directly synthesize 5-AVAB without the need for trimethyl-lysine to synthesize 5-AVA intermediates (40). 5-AVAB could take advantage of the methyl group in betaine for biosynthesis and, similar to betaine, conducted a pivotal role in the growth and development of the fetal nervous system (37). 5-AVAB has recently been demonstrated to be crucial in fetal tissue (41), claiming to be an important component of brain development, and its mechanism was related to promoting the growth of neural axons (17), which to some extent proves that this substance plays a key role as a mediator in the gut-brain axis.

Antibiotic-treated mice in our research had significantly lower levels of 5-AVAB and its metabolite TMAO in the hippocampus than normal mice, which indicated that 5-AVAB may be able to mediate the anti-postnatal depression effect. Interestingly, although both the fecal transplantation of PPD group and 919TJ group could increase the level of 5-AVAB in the hippocampus, the FS mice are still in a depressive state, indicating that the remission of postpartum depression was not only mediated by 5-AVAB. Compared with ABX+TJ group, 5-AVAB in hippocampus of FT group significantly increased and erucamide decreased, while only 5-AVAB in FS group increased, and erucamide of FT mice was significantly lower than that of FS. Therefore, we believe that 919 Syrup can reduce the ratio of erucamide to 5-AVAB by regulating intestinal flora, thereby alleviating postpartum depression.

Next, we searched for intestinal microflora that may synthesize erucamide and 5-AVAB through correlation analysis. 919 Syrup may downregulate the level of erucamide in hippocampus by upregulating Bacteroides. The downregulation of erucamide after fecal transplantation may be also related to Bacteroides. After antibiotic treatment, the level of 5-AVAB in the hippocampus of mice was significantly decreased, which may be related to the decrease of Lactobacillus, Ruminiclostridium, and Bacteroides. Transplantation of 919TJ feces could restore these bacteria and increase the level of 5-AVAB in hippocampus.

Proteobacteria and its subclasses such as Alpha-proteobacteria, Gamma-proteobacteria and Delta-proteobacteria, were upregulated in ABX+TJ mice compared with 919 TJ and FT. Proteobacteria growth has been suggested by researchers as a possible diagnostic marker for intestinal ecological disorders and disease risks (44). Studies have shown that Proteobacteria is associated with depressive behaviors (45–49). The abundance of Bacteroides, Proteobacteria, and Actinomycetes in the stool of depressive patients was considerably higher than that of the healthy group, according to a clinical trial that examined stool samples from 46 patients with major depression and 30 healthy volunteers (45). After chronic unpredictable restraint stress, the abundance of Proteobacteria in intestine of postnatal depression mice significantly increased compared with normal postnatal mice (50). The relative abundance levels of fecal Bacteroides generating GABA have been shown to be inversely connected with brain patterns associated with depression (51), consistent with our results. However, some studies revealed that Bacteroides species had a higher proportion in major depression disorder compared with healthy people (52, 53). One research results showed that transplantation of Bacteroides into antibiotic-treated mice induced depression-like behaviors and had an impairment on hippocampal neurogenesis (54). Lactobacillales, Lactobacillaceae and Lactobacillus were increased in 919TJ and FT compared with ABX+TJ. As a type of probiotic, there is a popular belief that Lactobacillus is beneficial to improve depression syndrome (55, 56). Consuming a Lactobacillus strain was said to control GABA receptor expression via the vagus nerves and decreased stress-induced corticosterone and depression-related behaviors (31).

However, there are still some shortcomings in this study, the most important of which is the verification of anti-postnatal depression functions of hippocampal metabolites like 5-AVAB and depression-inducing effects of erucamide, which is the most direct evidence supporting the key arguments of this study. The second step is to verify the causal relationship between specific bacteria and hippocampal metabolites. For example, there is a need to selectively detect the level of hippocampal metabolites after colonization of specific bacteria in intestinal tract of mice. Finally, the molecular mechanisms in central nervous system require to be further explored. That is to identify target cells or signal pathways at the gene or protein level after confirming the anti-postnatal depression effect of hippocampal metabolites.

**Figure 8:**
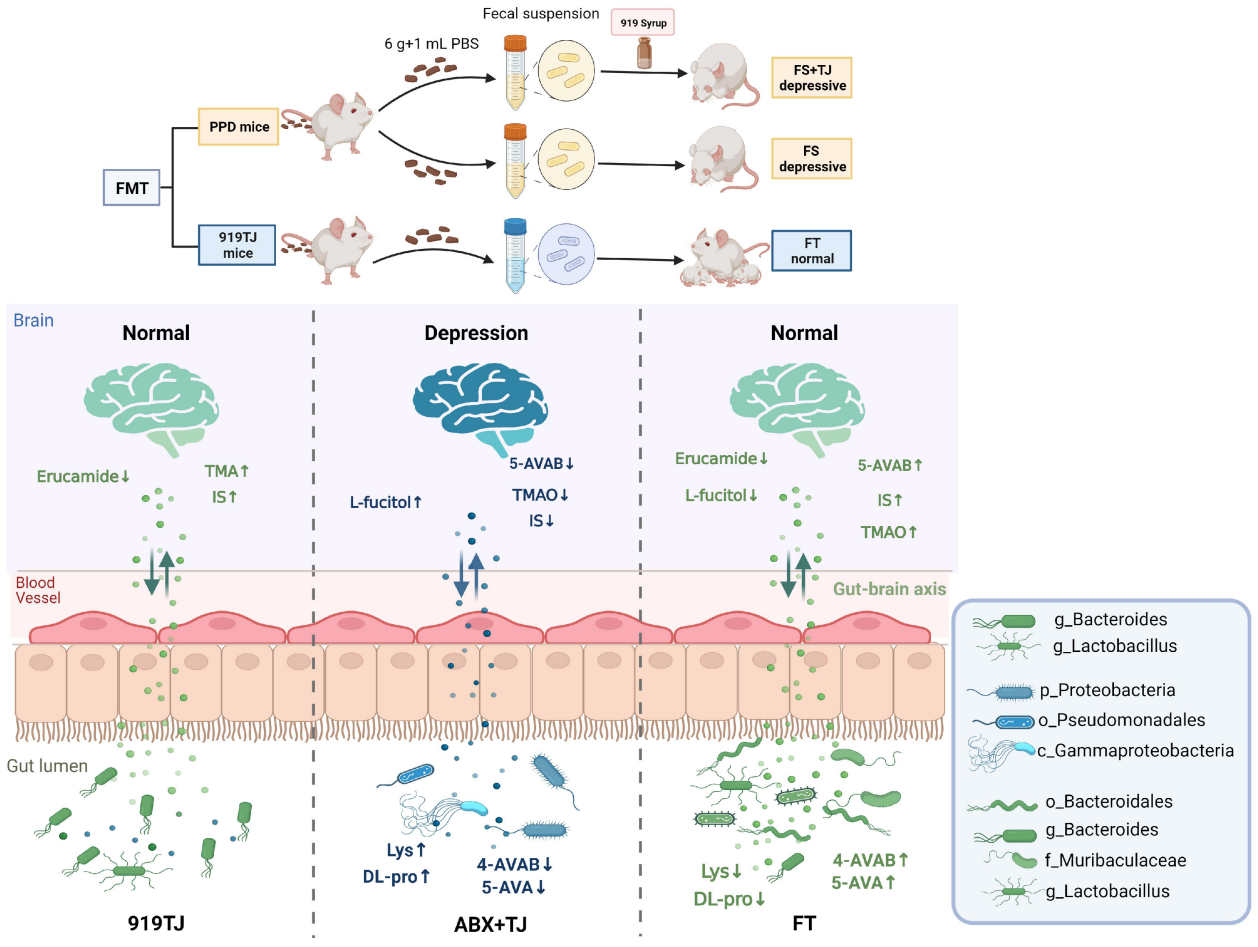
Mechanism map of 919 Syrup regulating intestinal flora in the treatment of postpartum depression.

In summary, our research preliminarily suggests that 919 Syrup may reduce the level of erucamide and increase 5-AVAB in hippocampus of mice by augmenting abundance of intestinal floras such as Bacteroides and Lactobacillus, thereby treating postpartum depression, laying a scientific foundation for future pathological research and development of therapeutic drugs for postpartum depression to a certain extent (**Figure 8**).

## Data availability statement

The 16s rDNA sequencing data are deposited in the NCBI SRA database (BioProject accession number: PRJNA949513). The metabolomics data of hippocampus have been deposited in the OMIX, China National Center for Bioinformation/Beijing Institute of Genomics, Chinese Academy of Sciences (Accession no: OMIX003710). Other data are available from the corresponding author upon reasonable request.

## Ethics statement

The animal study was reviewed and approved by Shanghai Public Health Clinical Center Laboratory Animal Welfare (2021-A046-01).

## Author contributions

Conception and design of study: QZ, SW, XT, WL and PG. Implementation of the experiment and acquisition of data: QZ, SW, XT and WL. Analysis and/or interpretation of data: QZ, SW, and PG. Drafting the manuscript: QZ and SW. Revising the manuscript critically for important intellectual content: QZ, SW, XT, WL and PG. Approval of the version of the manuscript to be published: QZ, SW, XT, WL and PG. All authors contributed to the article and approved the submitted version and agree to be accountable for the content of the work.

## Supplementary material

Supplemental Figure Correlation analysis between significantly differential hippocampal metabolites and faecal flora of FT vs ABX+TJ. (*: P<0.05; **: P<0.01; ***: P<0.001; n: P≥0.05).
